# Hybrid Functional Near-Infrared Spectroscopy System and Electromyography for Prosthetic Knee Control

**DOI:** 10.3390/bios14110553

**Published:** 2024-11-13

**Authors:** Nouf Jubran AlQahtani, Ibraheem Al-Naib, Ijlal Shahrukh Ateeq, Murad Althobaiti

**Affiliations:** 1 Biomedical Engineering Department, College of Engineering, Imam Abdulrahman Bin Faisal University, Dammam 34212, Saudi Arabia; no.ju.qi@gmail.com (N.J.A.);; 2 Bioengineering Department, King Fahd University of Petroleum & Minerals, Dhahran 31261, Saudi Arabia; ibraheem.naib@kfupm.edu.sa; 3 Interdisciplinary Research Center for Communication Systems and Sensing, King Fahd University of Petroleum & Minerals, Dhahran 31261, Saudi Arabia

**Keywords:** neurorehabilitation, electromyography (EMG), brain–computer interfaces (BCIs), functional near-infrared spectroscopy (fNIRS), motor imagery (MI), lower prosthetic limbs

## Abstract

The increasing number of individuals with limb loss worldwide highlights the need for advancements in prosthetic knee technology. To improve control and quality of life, integrating brain–computer communication with motor imagery offers a promising solution. This study introduces a hybrid system that combines electromyography (EMG) and functional near-infrared spectroscopy (fNIRS) to address these limitations and enhance the control of knee movements for individuals with above-knee amputations. The study involved an experiment with nine healthy male participants, consisting of two sessions: real execution and imagined execution using motor imagery. The OpenBCI Cyton board collected EMG signals corresponding to the desired movements, while fNIRS monitored brain activity in the prefrontal and motor cortices. The analysis of the simultaneous measurement of the muscular and hemodynamic responses demonstrated that combining these data sources significantly improved the classification accuracy compared to using each dataset alone. The results showed that integrating both the EMG and fNIRS data consistently achieved a higher classification accuracy. More specifically, the Support Vector Machine performed the best during the motor imagery tasks, with an average accuracy of 49.61%, while the Linear Discriminant Analysis excelled in the real execution tasks, achieving an average accuracy of 89.67%. This research validates the feasibility of using a hybrid approach with EMG and fNIRS to enable prosthetic knee control through motor imagery, representing a significant advancement potential in prosthetic technology.

## 1. Introduction

There are over 2.3 million individuals with limb loss in the United States, with a majority experiencing lower limb loss [1]. As limb loss increases due to factors like diabetes and traffic accidents, research efforts to restore limb function are crucial for rehabilitation and improving daily life for affected individuals [2]. Prostheses are artificial devices created to restore the function of lost limbs. There are generally two categories of lower limb prostheses: basic and advanced [3,4,5]. Basic prostheses lack advanced technology and provide only basic functionality and support, whereas advanced prostheses incorporate sophisticated technology to offer precise control [3]. The development of prosthetic limbs has a long history, dating back thousands of years [6,7]. The earliest known prosthetic device, an artificial big toe, was created around 710 BC by the Egyptians and was uncovered in 2000. More than 500 years later, advancements led to the creation of iron hands. The 19th century saw major progress in both limb removal surgery and prosthetic design. Initially, prostheses were primarily designed to support the body’s structure, rather than to restore limb function [7]. Between 1919 and 1999, body-powered prostheses made from metal and plastic materials were developed, marking the initial effort to restore limb function [6,7]. Advanced prostheses have been developed over the years, and since then, there have been remarkable advancements in rehabilitation technology. Today, the pinnacle of prosthetic technology includes advanced prostheses, which are motorized devices controlled by electrical signals from muscles or nerves located above the amputation site [6].

Earlier systems, which are integrated into prostheses, to provide individuals with limb loss some degree of control could not fully replicate the functionality of a natural limb. Consequently, there is significant interest in integrating motor imagery (MI) with electroencephalography (EEG) and functional near-infrared spectroscopy (fNIRS) technologies to enhance prosthetic control [8,9,10,11,12]. Motor imagery, which involves mentally stimulating movements, has become an effective method for improving prosthetic function. It is increasingly used to assist individuals with disabilities by boosting their motor capabilities and fostering independence. Numerous studies have explored ways to optimize the use of motor imagery for these individuals [12,13,14,15,16,17,18,19,20]. Motor imagery enables patients to mentally control prosthetic limbs like their own, which helps to reinforce the neural pathways for limb movement. It is worth mentioning that individuals with congenital limb differences who have experienced limb loss exhibit distinct neural activation patterns compared to those with typical limb function. After limb loss, the brain reorganizes to adapt to changes in the body schema, potentially engaging alternative neural pathways [21]. Although concerns persist about fNIRS’s ability to capture relevant data, previous studies have successfully demonstrated its capability in investigating motor imagery in individuals with limb loss [22,23].

Currently, various technologies are being integrated to achieve complete control over prostheses. For instance, combinations of EEG and electromyography (EMG), EEG and fNIRS, as well as EMG and fNIRS, are being developed to create more advanced prosthetic solutions for individuals with limb loss. Previously, fNIRS and EEG modalities have been employed to control prostheses through MI [23,24,25,26,27,28]. Generally, prosthetic limbs can be controlled using either single-modality or hybrid-modality approaches. Single-modality methods involve controlling the prosthesis with MI signals from either EEG or fNIRS. In contrast, hybrid-modality methods combine EEG with fNIRS or EMG with fNIRS to enhance control capabilities [29].

The EMG-fNIRS hybrid system is a recent development that has been utilized to control arm prostheses. The concept of integrating EMG and fNIRS techniques was first introduced in 2014 by Rea et al. [30], who investigated the relevance of fNIRS signals and motor preparation for lower limb rehabilitation. They collected fNIRS data from seven chronic stroke patients during the execution of left and right hip movements. Their analysis and classification demonstrated that fNIRS could effectively detect brain hemodynamic signals related to lower limb motor preparation in stroke patients. Since then, a few studies have explored this hybrid system’s feasibility in rehabilitation, with one notable study by Sattar et al. [31] focusing on controlling a prosthetic arm using EMG-fNIRS. Additionally, a clear synchronization method for combining EMG and fNIRS for prosthesis control has not yet been established. Three years later, Lima-Pardin et al. [32] explored a novel technique to assist patients with mobility impairments by combining fNIRS with biomechanical assessments during the initiation of assistive steps. Their findings were promising, suggesting that integrating fNIRS and biomechanical analyses could be highly effective. Additionally, Klerk et al. [33] employed EMG and fNIRS signals to investigate the spontaneous and unconscious tendency to imitate others’ behavior, a topic within the realm of social and cultural learning rather than rehabilitation. Despite this divergent application, they successfully validated their hypothesis using EMG and fNIRS signals.

A study by Caliandro et al. [34] aimed to explore the connection between lower limb kinematics and cortical gait control mechanisms in stroke patients. They recorded the prefrontal cortex activity using fNIRS and muscle activity with EMG in twenty-two patients during gait movements. The study found that tasks assisted by the Ekso exoskeleton increased the prefrontal activity, though more research is needed to further investigate this effect. Similarly, Wang et al. [35] examined muscular activity during motor tasks with EMG and fNIRS systems, using data from twenty-one healthy participants. They analyzed the non-linear features of EMG signals and fuzzy approximate entropy to assess motor task changes, while brain activity interactions were analyzed using effective connectivity, graph theory metrics, and functional connectivity. Their findings confirmed a relationship between brain and muscular activity across various motor tasks. Another study by Daniel et al. [36] investigated the correlation between EMG and fNIRS signals during dynamic movements. Data from five female participants supported their hypothesis that changes in EMG and fNIRS signals are interrelated during dynamic movements, with a positive correlation observed in all the participants. Syed et al. [22] developed a new technique using EMG and fNIRS to interpret upper limb motions, achieving an average accuracy of 94.5% across eight different motions, demonstrating the technique’s effectiveness for prosthetic arm control. Similarly, Sattar et al. [31] introduced a hybrid brain–machine interface combining EMG and fNIRS to overcome limitations of myoelectric upper limb prostheses, achieving a high average accuracy and validating the hybrid system’s potential to enhance the control of multifunctional upper limb prostheses.

This research will pioneer the use of a hybrid EMG-fNIRS system, which merges the advantages of both EMG and fNIRS technologies. This integration intends to draw on the advantages of both technologies to control smart prosthetic limbs, an area that has seen limited exploration, especially for lower extremities. By combining EMG and fNIRS technologies, the study seeks to provide a novel method for controlling prosthetic knees through motor imagery, aiming to offer individuals with lower limb loss a level of control akin to that of natural limbs. To the best of our knowledge, no existing studies have investigated this EMG-fNIRS-based MI approach for prosthetic knee control, making this research a significant step toward overcoming current prosthetic challenges and improving the quality of life for individuals with limb loss.

## 2. Methods

**Figure 1:**
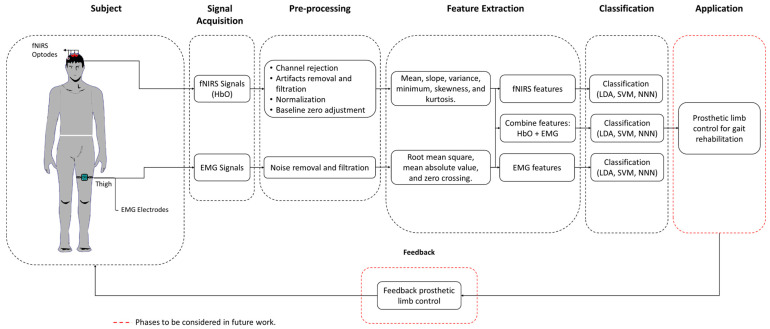
The experimental framework: sEMG electrodes are placed on the thigh, and fNIRS optodes are positioned on the head. The acquired HbO and EMG signals are pre-processed, and features are extracted from both types of data. These features are used in classifiers to differentiate between knee movements, with system feedback aiding in refining control in future phases.

In this study, muscle and brain hemodynamic signals were recorded simultaneously. Muscle activity was measured non-invasively from the muscles responsible for knee movements using surface EMG (sEMG) electrodes, while brain hemodynamics was measured from the prefrontal and motor cortex regions using a continuous wave fNIRS system. Following the simultaneous recording, the physiological signals were pre-processed and then the features were extracted for classification. These features were used to categorize knee movements as either extension or flexion. Figure 1 summarizes the overall experimental framework for this study.

### 2.1. Optode and EMG Electrode Placement

**Figure 2:**
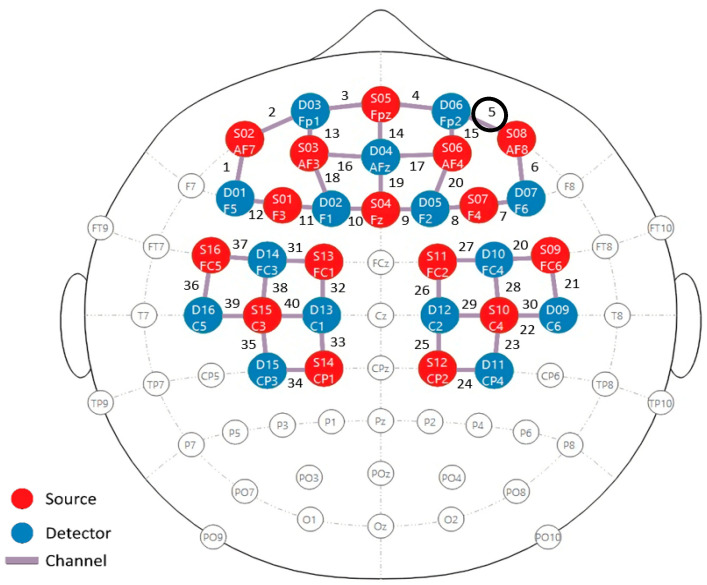
Placement of fNIRS optodes on the prefrontal and motor cortexes in a 16 × 15 montage.

To accurately measure the desired signals, fNIRS optodes and sEMG electrodes must be precisely positioned. To capture brain activity in the regions of interest, the optodes were placed on the prefrontal and motor cortex areas [17], as shown in Figure 2. A total of sixteen sources and fifteen detectors (16 × 15) were positioned on the right and left hemispheres of the prefrontal and primary motor cortex. The montage was designed based on the international 10–20 system for EEG electrode placement [37]. This montage results in 40 channels for data acquisition, with distances ranging from 2.9 cm to 3.1 cm between each emitter and detector [37,38].

**Figure 3:**
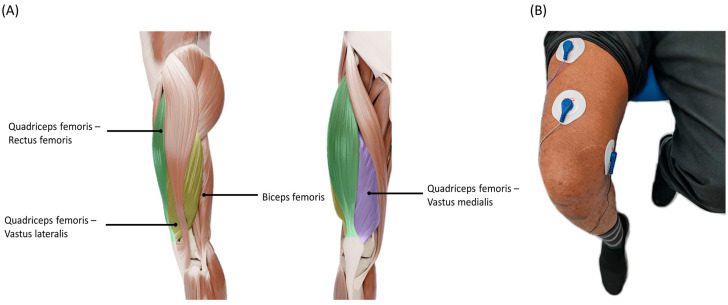
(**A**) Placement of sEMG electrodes on targeted muscles and (**B**) placement of sEMG electrodes on a participant.

EMG signals related to knee extension and flexion were recorded using sEMG electrodes. Five electrodes were utilized in this study: (i) two for the quadriceps femoris, (ii) two for the hamstrings, and (iii) one reference electrode placed on the bony structure of the knee, as suggested in [39]. Figure 3 shows the electrode placement, determined after multiple trials to ensure accurate positioning on the targeted muscles.

### 2.2. Sending Triggers and Synchronization Unit

**Figure 4:**
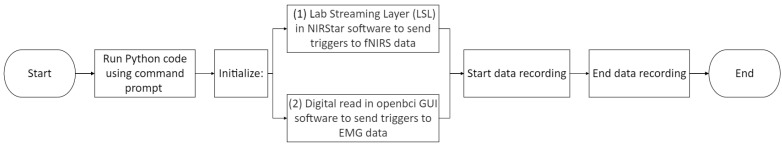
Illustration of simultaneous measurement workflow of Python algorithm.

To ensure precise simultaneous measurement of EMG and fNIRS signals, an algorithm was developed using Python software (version 3.8) to operate both systems and manage stimuli presentation. This algorithm provides a full-screen display for the experiment. Figure 4 depicts an overview of the algorithm’s workflow.

**Figure 5:**
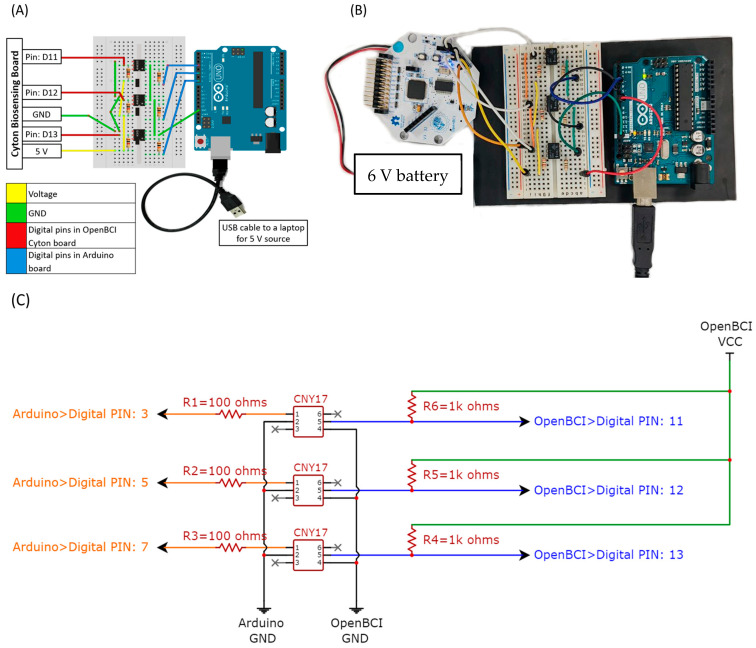
(**A**) The circuit connection diagram for the synchronization unit, (**B**) an image of the circuit connection, and (**C**) a schematic diagram for the synchronization unit.

The Lab Streaming Layer (LSL) is a method that enables real-time data acquisition and time-synchronized streaming across multiple modalities. It has proven to be highly effective for streaming fNIRS data and managing event triggers [40]. Conversely, EMG triggers were controlled using an external circuit, as illustrated in Figure 5. The circuit was assembled with three optocouplers, three 100 Ω resistors, three 1 KΩ resistors, an Arduino UNO board, and a Cyton biosensing OpenBCI board. The first optocoupler sends a trigger at the start of the rest event, the second at the beginning of the knee extension event, and the third at the knee flexion event. Figure 5 provides a detailed schematic of the circuit connections. The circuit was designed to send triggers to EMG signals through digital switching, indicating the output shifts from HIGH (1) to LOW (0) when a 5 V external trigger is applied to the input anode.

### 2.3. Experimental Protocol and System Setup

The brain activity in the prefrontal and motor cortex regions was measured using a continuous wave fNIRS system (NIRSPORT 1, manufactured by NIRx Medical Technologies, LLC, Berlin, Germany), which operates at wavelengths of 760 nm and 850 nm and samples data at a rate of 3.47 Hz. Previous fNIRS research has shown significant hemodynamic activation in these brain regions during motor activities [17,18,31,34,41,42]. These studies confirmed that the prefrontal region and primary motor cortex play crucial roles in the planning, initiation, and execution of intended movements. Anatomically, these findings are consistent with their involvement in the gait cycle [43].

The muscular activity of the quadriceps femoris and hamstring muscles was measured using sEMG electrodes connected to a Cyton biosensing OpenBCI board, an Arduino-compatible device with an 8-channel bio-amplifier and a 32-bit processor. The Cyton board sampled muscle activity at 250 Hz per channel and communicated wirelessly with a laptop via the OpenBCI USB dongle for data streaming and recording. Electrodes for the quadriceps femoris were connected to the top N1P and bottom N1P pins, while electrodes for the hamstrings were connected to the top N3P and bottom N3P pins. The reference electrode was connected to the bottom BIAS pin. EMG data from the quadriceps femoris were assigned to channel #1 on the OpenBCI GUI software (version v5.2.2), and data from the hamstrings were assigned to channel #3. As stated previously, the placement of sEMG electrodes follows the guidelines in [39] and the connection was as previously shown in Figure 5.

**Figure 6:**
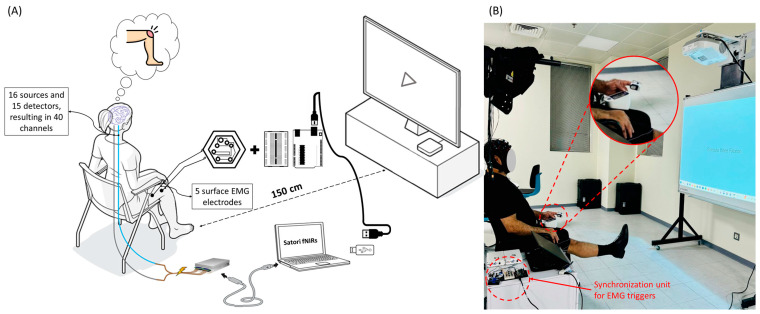
(**A**) Experimental setup diagram and (**B**) a photo of the experimental setup.

Figure 6 provides a basic overview of the experimental setup. The experiment was divided into two sessions: a training session and a testing session. During the training session, participants performed specific tasks in a controlled environment to minimize external disturbances. Each participant sat in a comfortable chair and was instructed to remain relaxed to avoid unnecessary movements or distractions. The tasks were displayed on a large screen positioned approximately 150 cm from the participant.

### 2.4. Experimental Paradigm, Stimuli, and Procedure

**Figure 7:**
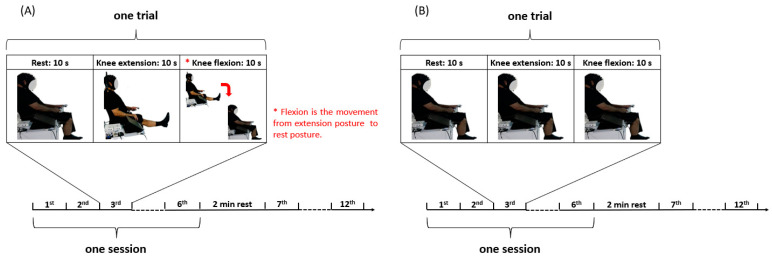
Experimental paradigm (**A**) for real execution of knee movements and (**B**) for the decision to execute knee movements without real execution.

Initially, participants were asked to relax for at least 5 min before starting the experiment and to continue maintaining relaxation throughout. Figure 7 illustrates the experimental procedure designed for this investigation. The experiment was conducted in two phases: (i) real execution (RE) of the task and (ii) imagined execution of the task through MI. The procedure began with the first phase, where participants physically completed the task by following written and auditory commands displayed on the screen. This phase consisted of twelve trials, divided into two sub-sessions (6 trials each), with a two-minute break between them. Each trial started with a 10 s pre-rest period during which participants remained calm. Following this, participants executed knee extension for 10 s, followed by knee flexion for an additional 10 s. This process was repeated twelve times for each participant, as shown in Figure 7A. This indicates that the duration of both datasets is 480 s for each participant. After a five-minute break, participants proceeded to the second phase, in which they stimulated the task mentally, without physically performing the task, as illustrated in Figure 7B.

Nine healthy male participants (mean age: 46.56 ± 12.59) participated in the experiment. Among them, eight were right-handed, and one was left-handed. Identifying handedness is crucial for determining which leg is included in the experiment and where to place the sEMG electrodes. For example, if a participant is right-handed, the sEMG electrodes are positioned on the right leg. Before the experiment started, physiological parameters such as blood pressure (BP), heart rate (HR), oxygen saturation (SpO_2_), weight, and height were recorded. We conducted measurements of BP and HR using a digital sphygmomanometer, while SpO_2_ was assessed through a pulse oximeter. Participants were briefed on the experimental protocol and provided written consent before proceeding. They also received a brief instructional session on knee movements prior to the experiment. The study was approved by the Institutional Review Board and the Ethical Committee at Imam Abdulrahman bin Faisal University (IAU).

### 2.5. Signal Acquisition and Pre-Processing

In MATLAB^®^ software (version 24a), the EMG signals were first processed using a fourth-order Butterworth bandpass filter (BPF) with a frequency range of 1 Hz to 30 Hz, along with a notch filter with a frequency range of 59 Hz to 61 Hz. The BPF was applied for baseline correction to address drift caused by issues such as improper electrode placement, which can affect impedance and introduce low-frequency noise. Other factors contributing to drift include temperature changes, participant movement, and variations in the instrumentation and amplifiers. This BPF was chosen to eliminate low-frequency artifacts while retaining the essential frequencies of the EMG signal, which range from 50 Hz to 150 Hz. The notch filter was then used to further refine the signal and remove power line interference [44,45]. After filtering, the data were segmented into 10 s intervals. The time representation of the EMG channels was subsequently assessed before extracting the selected features.

**Figure 8:**
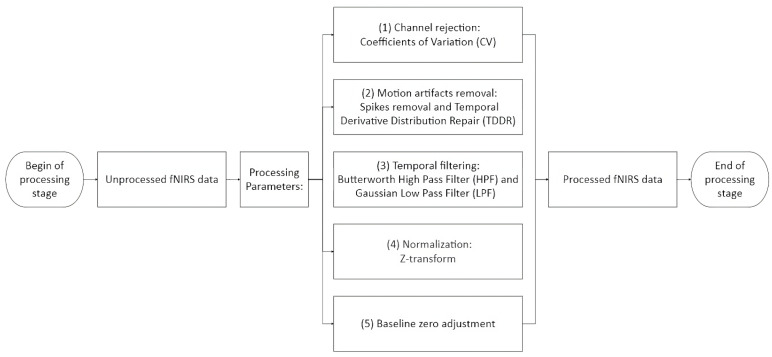
Overview of the pre-processing steps applied to the fNIRS data.

In our study, the methodologies employed have been carefully selected based on established practices in fNIRS research to enhance HbO signals and reject extracerebral noise. We implemented several well-documented techniques, including temporal filtering, Temporal Derivative Distribution Repair (TDDR), and z-normalization, all of which are effective in isolating neural signals. For pre-processing the acquired fNIRS data, we utilized Satori^®^ software (version 2.0), developed by Brain Innovation B.V., which is specifically designed for analyzing fNIRS signals and effectively eliminates noise while filtering the data [35]. It provides the latest standard processing techniques and statistical tools for fNIRS analysis. Figure 8 outlines the procedures for the pre-processing of fNIRS data.

To evaluate signal quality, the Coefficient of Variation (CV), defined as the ratio of the standard deviation to the mean, was employed. Channels with a CV exceeding 10% were excluded from analysis due to unphysiological noise, such as motion artifacts, power line interference, optode–skin interface issues, and ambient light interference [46]. Subsequently, brief disturbances with broad frequency ranges, known as spikes, were addressed using a specialized technique. This method involves detecting spikes with a reliable algorithm that requires four parameters: iteration (n = 10), lag (t = 5 s), threshold (th = 3.50), and influence (i = 0.50) [47]. These parameters enable the algorithm to effectively identify and manage spikes, which were subsequently corrected using the default monotonic interpolation method. Moreover, the TDDR technique was applied to correct motion artifacts in the fNIRS data. This method employs a reliable regression to correct baseline shifts and spike artifacts [48]. To further enhance noise reduction, temporal filters were applied: a second-order Butterworth high-pass filter (HPF) with a 0.01 Hz cutoff frequency and a second-order Gaussian low-pass filter (LPF) with a 0.4 Hz cutoff frequency [49]. To address the variability in signal levels between channels caused by physical and physiological factors, baseline z-normalization was applied to the fNIRS data. This normalization improved comparability across channels and participants. Finally, a baseline-zero adjustment was performed, shifting the baseline of the fNIRS signal by a percentile of the overall signal level, with a default shift of 5% to preserve the signal’s overall structure [49].

### 2.6. Feature Extraction and Classification

Once the fNIRS data were pre-processed, they were imported into MATLAB^®^ for further analysis. Information and features were extracted from the EMG and fNIRS datasets to facilitate the classification process, which could potentially enhance existing prosthetic knee technologies. Including both execution and MI sessions for each participant helps address the typically lower amplitude of EMG signals observed during MI sessions. By comparing data from these two sessions, significant variations in the EMG signals can be detected, even in the absence of strong muscle contractions. This is because MI can still generate low-amplitude EMG signals due to neural impulses affecting the muscles, even without direct physical movement [50,51].

Three time-domain features were derived from EMG segments: root mean square (RMS), mean absolute value (MAV), and zero crossing (ZC). These features are commonly used in real-time EMG signal analysis due to their relatively low computational requirements [52]. Previous studies have demonstrated that extracting features from HbO signals is more effective for classification than using HbR or total hemoglobin [16]. Consequently, this study utilizes HbO data to extract six distinct statistical features: Signal Slope (SS), Signal Mean (SM), Signal Variance (SV), Signal Skewness (SW), Signal Kurtosis (SK), and Signal Minimum (SN). These features were selected as recommended in [16].

Features extracted from all channels in each dataset (EMG, fNIRS, and combined EMG + fNIRS) were used for classification. Common classification techniques for fNIRS and EMG systems include Linear Discriminant Analysis (LDA), Support Vector Machine (SVM), and Narrow Neural Network (NNN) [49,53,54,55]. The MATLAB^®^ Classification Learner app was used to evaluate classification accuracies for resting, knee extension, and knee flexion tasks across nine participants. Classification performance was assessed using 10-fold cross-validation.

## 3. Results

### 3.1. Hemodynamic Responses and Muscle Activity

**Figure 9:**
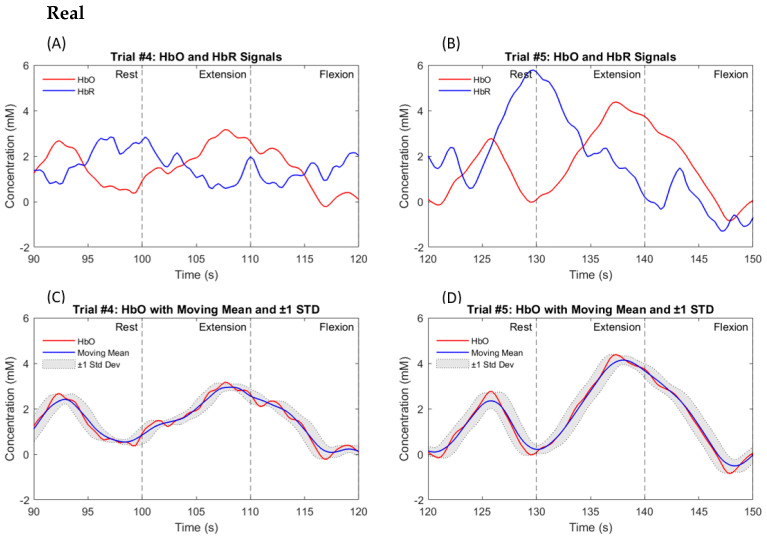
A typical hemodynamic response for real knee extension and knee flexion tasks in participant #4, a 42-year-old healthy male, is depicted. Dashed black lines indicate the start and end of the task period. (**A**) shows the HbO (in red) and HbR (in blue) for channel #5 across one trial, while (**C**) presents the mean and STD of the HbO signal for that trial. (**B**,**D**) display similar information for a second trial.

**Figure 10:**
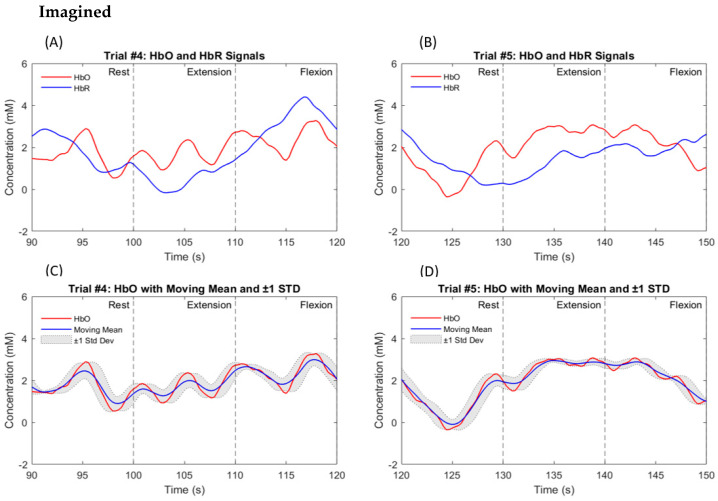
A typical hemodynamic response for imagined knee extension and knee flexion tasks in participant #4, a 42-year-old healthy male, is depicted. Dashed black lines indicate the start and end of the task period. (**A**) shows the HbO (in red) and HbR (in blue) for channel #5 across one trial, while (**C**) presents the mean and STD of the HbO signal for that trial. (**B**,**D**) display similar information for a second trial.

**Figure 11:**
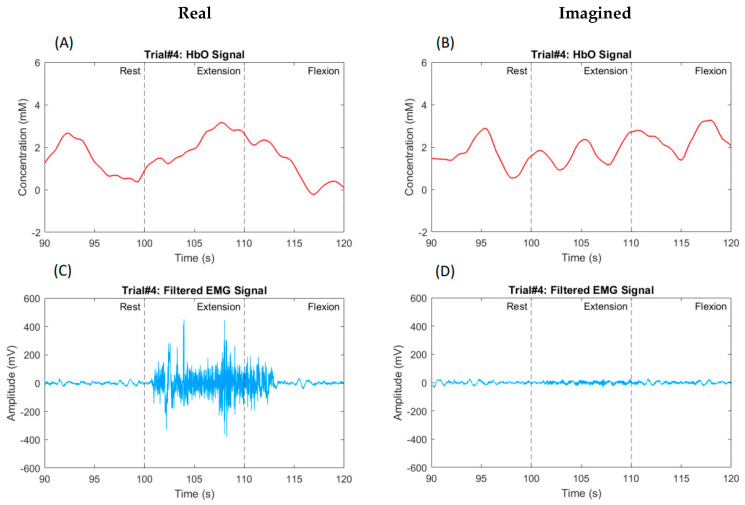
The hemodynamic response and EMG signal for real (on the left side) and imagined (on the right side) knee extension and knee flexion tasks in participant #4, a 42-year-old healthy male, are depicted. Dashed black lines indicate the start and end of the task period. (**A**) shows the HbO (in red) for channel #5 across one trial during the real experiment, while (**C**) presents the EMG signal for the same trial. (**B**,**D**) display similar information for the same trial but during the imagined experiment.

As stated before, a total of nine participants were enrolled in this study, all of whom were in good health and had a high level of education. The hemodynamic response was measured in the left and right hemispheres of the prefrontal and motor cortices in nine participants. Data from all participants clearly show the HbO response triggered by both the actual and imagined knee extension and knee flexion tasks. Figure 9A,B illustrate the hemodynamic response for participant #4, a 42-year-old healthy male. Figure 9 and Figure 10 represent the responses to the knee extension and knee flexion tasks in the real and imagery experiments, respectively. Channel #5, highlighted in Figure 2, demonstrated a clear hemodynamic response during these tasks. The HbO and HbR data from channel #5 for two trials are shown in Figure 9 and Figure 10. Despite some variations, other channels exhibited similar trends. Figure 9C,D present the mean and standard deviation (STD) of the HbO signal for the same participant during real tasks, while Figure 10C,D show these metrics for imagined tasks. Figure 11 displays the HbO and EMG signals for the real and imagined knee extension and knee flexion tasks for the same participant.

### 3.2. Classification Results Among Participants

The classification accuracies for both the real and imagined sessions across all the participants are presented in Table 1. Features extracted from each participant’s data were input into classifiers, and the corresponding accuracy was calculated for each individual participant.

### 3.3. Classification Results of Group

**Figure 12:**
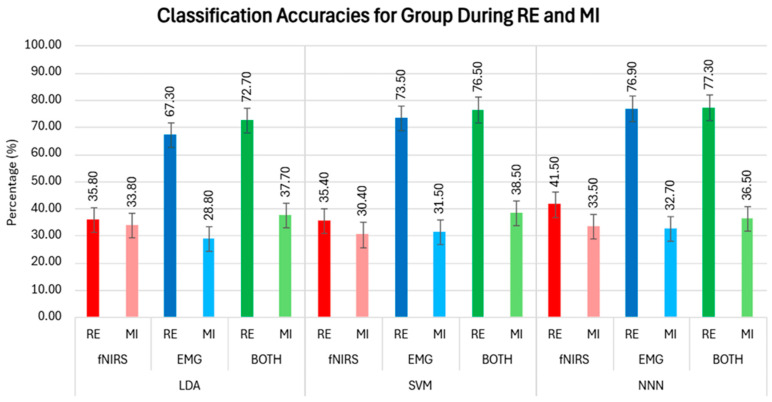
The classification accuracies for real (RE) and imagined (MI) tasks are illustrated with red shades for fNIRS data, blue shades for EMG data, and green shades for combined EMG and fNIRS data, with darker shades representing RE and lighter shades representing MI.

The group classification accuracy was calculated by organizing the data from all the participants into a single matrix and using this dataset as the input for each classifier. The classification accuracies for both the real and imagined sessions for all the participants are illustrated in Figure 12.

## 4. Discussion

This study showcased the potential of combining EMG and fNIRS signals to improve the classification accuracy and enhance the performance of prostheses in clinical applications. This integration overcomes the limitations inherent in each individual modality. The primary aim of this research was to introduce a new approach to control prosthetic knees using a non-invasive hybrid system. This approach focused on the two fundamental knee movements, knee extension and knee flexion, which are essential for maintaining functionality in individuals with above-knee amputation.

The proposed system produces two control commands based on EMG and fNIRS data collected from the prefrontal and motor areas and the quadriceps femoris and hamstring muscles. Remarkably, our findings revealed distinct HbO responses and muscular activation in all the participants during both the real and imagined knee extension and knee flexion exercises. Figure 9 and Figure 10 demonstrate that the knee extension task induced a higher concentration of HbO compared to the flexion task. This trend is consistent across all trials for all nine participants, as shown in Figure 9C,D and Figure 10C,D. These observations align with those from previous studies [13,16,17], which indicate that the lower fNIRS signal during MI, compared to the actual movement, is due to decreased neural activation, minimal muscle engagement, a less pronounced hemodynamic response, and lower cognitive demand during mental rehearsal.

Specifically, channel #5 exhibited the largest hemodynamic responses in both real and imagined experiments for all the participants. Since channel #5 is located in the prefrontal cortex, this observation could be related to its role in coordinating and executing motor actions, as it is involved in setting goals, making decisions, driving motivation, and managing cognitive control [56,57]. A possible explanation for the higher brain activation during the transition from rest-to-extension compared to extension-to-flexion could be attributed to the transient impact of posture. Both the EMG and fNIRS signals are influenced by changes in posture rather than by the specific tasks themselves.

To enhance clarity and impact, we unified the graph scales in the plots of the HbO levels and EMG signals during the real and imagined tasks, ensuring consistency across all the graphs. We calculated the average values of the signals collected during the real and imagined tasks to accurately identify the percentage differences between the EMG signals and fNIRS signals for both states. Notably, our analysis revealed that when the participants performed the real tasks, their HbO levels were significantly twice as high compared to when they imagined performing those same tasks. While this significant difference may not be visually apparent in Figure 9 and Figure 10, its importance was clearly reflected in the classification results. This indicates that the brain is more engaged and uses more oxygen when the participants physically perform the movements. Additionally, the EMG signal demonstrated an astonishing fifteen-fold increase during RE compared to MI. This discrepancy is anticipated due to the continuous muscle stimulation during real movements, which is absent in imagined tasks.

The proposed system was assessed using feature extraction and classification techniques. Effective feature extraction is essential for attaining a high classification accuracy. In this study, nine commonly used statistical features were extracted from both EMG and fNIRS datasets to classify events. The performance of the LDA, SVM, and NNN classifiers was evaluated to differentiate between the resting, extension, and flexion tasks using these features. The results in Table 1 demonstrated that incorporating information from both the brain and muscles significantly improves the classification accuracy compared to using each source of data individually. Initially, the system’s performance was evaluated using data from each participant, and it was found that combining the data consistently achieved a higher classification accuracy than using each type of data alone. This outcome was expected, as having more and varied yet related information helps to better differentiate between tasks. The SVM consistently outperformed the other classifiers during MI, as shown in Table 1, in which its average accuracy was 49.61%. However, the LDA demonstrated a superior performance during the RE tasks, achieving an average accuracy of 89.67%.

Ultimately, the system’s performance was assessed using data from all the participants combined into a single matrix, and the results displayed a trend similar to those observed when analyzing data from each individual participant separately. Figure 12 presents these findings, showing that the SVM demonstrated the best performance during the MI, while NNN achieved the highest results during the RE tasks. The reduced accuracy in the imagery experiments compared to the real experiments could be due to the weaker EMG and fNIRS signal strength during the imagery tasks. This implies that further analysis, an increased number of participants, or additional trials may be needed to enhance the signal-to-noise ratio and improve the accuracy. Moreover, fluctuations in the timing of the task routine during imagery trials might disrupt the consistency of statistical features, potentially resulting in a lower classification accuracy.

Generally, there are several factors contribute to the low classification accuracy during motor imagery. First, the inherent variability in brain activity during motor imagery tasks can lead to inconsistent neural patterns, making it difficult for classification algorithms to reliably distinguish between different imagined movements. Second, individual differences in participants’ familiarity with motor imagery and their cognitive strategies can further complicate the classification process. Third, unifying the sampling rate and utilizing short separation channels could significantly improve classification accuracy. Therefore, addressing these factors should be a priority in future studies to enhance the reliability of motor imagery classification.

Previous research [58] has shown that incorporating extra features or electrodes can substantially boost recognition rates, achieving around 95% accuracy. However, it is crucial to recognize that even this level of accuracy may fall short for critical applications like hand and leg prostheses. A 5% error rate translates to one incorrect decision for every twenty attempts, which is unacceptable for hand control, where precision is vital. This concern is even more pronounced for leg prostheses, as minor failures can result in falls and serious injuries. Therefore, our study underscores the urgent need for ongoing advancements in accuracy and reliability to ensure the safety and effectiveness of prosthetic devices. Additionally, classification accuracies in individuals with limb loss participants often fall short compared to healthy individuals due to factors like variations in muscle activation patterns, differences in residual limb anatomy, and the complexities of dynamic signal acquisition. Addressing these challenges is vital for developing effective classification systems tailored to the needs of individuals with lower limb loss. By employing advanced pre-processing techniques and improved algorithms, we can significantly enhance the classifier performance, ultimately leading to better outcomes for users.

Many studies and reviews have explored and discussed the use of EMG/fNIRS to enhance lower prosthetic limbs [23,28,59,60,61,62]. However, prior to this study, only a limited number of studies have investigated the correlation between EMG and fNIRS data during dynamic movements. In the study by Sattar et al. [31], the hybrid brain-machine interface was used to enhance upper limb prostheses. Tested on fifteen healthy individuals and three individuals with transhumeral limb loss, the system showed a high accuracy: 94.6% for elbow and 74% for wrist motions with EMG in the healthy participants, and 96.9% for hand motions with fNIRS. For the individuals with limb loss, the EMG accuracy was 74% for elbow and wrist motions, and the fNIRS accuracy was 94.5% for hand motions. The results suggest that combining these technologies improves the control and performance of upper limb prostheses for individuals with transhumeral limb loss. The main limitation of their system is that EMG and fNIRS signals are measured separately without synchronization, which is a crucial step for any hybrid system.

The limitations of this study include a small sample size, the absence of female participants, and significant age variability among the participants. Although the sample size is small, it is comparable to that used in other studies [16,41,63,64]. Despite these limitations, the results were promising. Each participant completed twelve trials of extension and twelve trials of flexion, resulting in a total of 108 datasets for each condition in both the real and imagery experiments. The findings suggest that motor imagery (mental rehearsal of movements) influences muscle activity in several ways. It activates brain regions involved in movement planning and execution, such as the primary motor cortex and premotor areas, leading to increased neural readiness that can affect muscle activity even without physical movement. Despite the absence of actual movement, motor imagery can induce minimal muscle activity, often referred to as “phantom” or “covert” activation, which can be detected using EMG. Repeated motor imagery training can enhance motor skills and muscle coordination by reinforcing neural pathways, leading to improved performance [65,66]. Additionally, it prepares muscles for action by activating the relevant neural circuits, resulting in faster and more efficient muscle activation during real movements. In rehabilitation, motor imagery can aid patients in restoring muscle function and coordination, particularly when physical movement is limited, and it plays a vital role in promoting motor learning and recovery [14,66,67]. In the future, fNIRS technology should be optimized to address some significant practical challenges, primarily the need for users to constantly wear a full-head cover cap. This requirement can impede daily activities and limit user acceptance, making it an impractical solution for long-term use. Furthermore, future research should consider modifications to fNIRS methodology to enhance sensitivity for this population. This study took vital steps toward understanding and developing a more compact, user-friendly fNIRS system that can be seamlessly integrated with EMG. By prioritizing the design of wearable systems that maximize comfort and usability, we aim to eventually overcome these limitations and provide more effective and accessible solutions for individuals using lower limb prostheses.

## 5. Conclusions

In this study, we explored the feasibility of decoding two distinct commands from the prefrontal cortex and motor cortex and the quadriceps and biceps femoris muscles using a hybrid EMG-fNIRS system, which combines the benefits of EMG and fNIRS modalities. This integration aims to leverage the strengths of both technologies to enhance clinical applications and rehabilitation. We simultaneously recorded muscle and brain signals during knee extension and flexion with nine healthy male participants performing both real and imagined movements. The collected data were carefully cleaned and filtered to eliminate noise and artifacts, followed by the extraction of relevant features for classification. The performance evaluations at both the individual and group levels consistently showed that using combined brain and muscle data resulted in an improved classification accuracy compared to relying on data from just one source. Specifically, the SVM classifier achieved the highest accuracy for MI tasks, with an average of 49.61%, while the LDA classifier was most effective for RE tasks, achieving an accuracy of 89.67%. These findings indicate that a hybrid EMG-fNIRS approach is viable for enhancing prosthetic knee control through mental imagery, marking a significant advancement in prosthetic technology by improving both functionality and control. Nevertheless, further research is necessary to expand the participant pool to include a more diverse sample and to optimize feature extraction techniques and classification algorithms. This will help validate the system’s effectiveness across different demographics and improve its generalizability. Additionally, future work should explore the integration of adaptive algorithms to refine prosthetic control and maximize the hybrid system’s performance. Overall, this study provides a robust foundation for advancing prosthetic knee technology and offers promising directions for future research aimed at enhancing the adaptability and functionality of prosthetic devices for a broader range of users, ultimately contributing to more effective and responsive prosthetic solutions.

## Figures and Tables

**Table 1:** Classification accuracies for both real and imagined sessions are provided for nine participants, with calculations performed for each individual. The highlighted values represent the highest accuracies for each case.

Classifier	Session	Data	S1	S2	S3	S4	S5	S6	S7	S8	S9	Average
LDA Accuracy (%)	RE	fNIRS	56.7	36.7	43.3	40	56.7	43.3	36.7	36.7	43.3	43.71
		EMG	76.7	86.7	86.7	93.3	76.6	80	96.67	66.7	66.7	81.12
		BOTH	83.3	90	93.3	97.1	93.3	90	96.7	86.7	76.6	89.67
	MI	fNIRS	33.3	33.3	30	26.7	26.7	30	33.3	46.7	36.7	32.97
		EMG	50	43.3	23.3	26.7	23.3	36.7	26.7	63.3	36.7	36.67
		BOTH	53.3	46.7	43.2	30	43.3	50	43.3	66.7	50	47.39
SVM Accuracy (%)	RE	fNIRS	60	46.7	46.7	56.7	43.3	43.3	36.7	43.3	46.7	47.04
		EMG	73.3	90	90	93.3	80	90	96.67	66.7	66.7	82.96
		BOTH	83.3	93.3	93.3	94.1	90	96.7	96.7	73.3	70	87.86
	MI	fNIRS	40	23.3	30	26.7	16.7	33.3	36.7	36.7	36.7	31.12
		EMG	53.3	36.7	26.7	30	20	23.3	33.3	60	20	33.70
		BOTH	70	50	43.2	43.3	36.7	43.3	46.7	70	43.3	49.61
NNN Accuracy (%)	RE	fNIRS	53.3	43.3	43.3	63.3	46.7	40	43.3	33.3	50	46.28
		EMG	56.7	83.8	86.7	93.3	76.7	86.7	96.67	60	63.3	78.21
		BOTH	66.7	86.7	93.3	97.1	86.7	93.3	96.7	66.7	70	84.13
	MI	fNIRS	36.67	23.3	30	23.3	20	23.3	23.3	36.7	46.7	29.25
		EMG	40	26.7	30	30	26.7	30	20	33.3	30	29.63
		BOTH	63.3	33.3	36.7	40	30	50	33.3	66.7	50	44.81

## Data Availability

The raw data supporting the conclusions of this article will be made available by the authors on request.
